# Combining a Hudl App With Telehealth to Increase Home Exercise Program Adherence in People With Chronic Diseases Experiencing Financial Distress: Randomized Controlled Trial

**DOI:** 10.2196/22659

**Published:** 2021-03-18

**Authors:** Ann Van de Winckel, Tanjila Nawshin, Casey Byron

**Affiliations:** 1 Division of Physical Therapy, Division of Rehabilitation Science Department of Rehabilitation Medicine, Medical School University of Minnesota Minneapolis, MN United States; 2 Division of Rehabilitation Science Department of Rehabilitation Medicine, Medical School University of Minnesota Minneapolis, MN United States; 3 Hennepin County Medical Center Minneapolis, MN United States

**Keywords:** chronic disease, spinal cord injury, stroke, telehealth, telemedicine, traumatic brain injury

## Abstract

**Background:**

Patients with chronic diseases often need to adhere to long-term individualized home exercise programs (HEPs). Limited adherence to long-term exercise given during physical therapy (PT) visits reduces the capacity of exercise to manage or improve symptoms related to chronic disease. In addition, a lower socioeconomic status negatively impacts exercise adherence. To mitigate this, apps that motivate people to exercise could be a viable option. Using an app through telehealth may help adults with chronic diseases to achieve long-term HEP adherence. However, because apps for rehabilitation are an emerging field, the feasibility of the app needs to be evaluated.

**Objective:**

To address HEP adherence in participants with chronic diseases who are experiencing financial distress, we aim to evaluate the feasibility of and satisfaction with the Hudl Technique app and telehealth and satisfaction with PT care and to monitor HEP adherence and compliance (ie, percentage of participant-recorded videos sent) in participants using the app with telehealth compared with those using standard HEPs on paper.

**Methods:**

We recruited patients scheduled for outpatient PT. We performed a randomized controlled trial in which the experimental group received weekly HEP demonstrations through app videos on a tablet with feedback on their self-recorded HEP video performance from the telehealth physical therapist. The control group received HEPs on paper without feedback, as is customary in PT practice. Demographic, clinical, and health coverage information was collected for screening and baseline measurements. Adherence and compliance were evaluated. Both groups completed surveys at 8 and 24 weeks on their satisfaction with PT care, and the experimental group also completed a survey on their satisfaction with the app with telehealth use. Descriptive and nonparametric statistics were used for within-group and between-group comparisons and analyzed with JMP, version 13.

**Results:**

Overall, 45 adults with chronic diseases who were experiencing financial distress were randomized into experimental (23/45, 51%) and control (22/45, 49%) groups, with 74% (17/23) and 86% (19/22) participants completing the 24-week HEP, respectively. The experimental group had an HEP adherence frequency of 4 (SD 2) to 5 (SD 2) times per week at 8 and 24 weeks (*P*=.14), whereas HEP adherence decreased in the control group from 4 (SD 2) to 3 (SD 2) times per week (*P*=.07), with a significant difference (*P*=.01) between groups at 24 weeks. Of the total participants, 68% (15/22) sent videos. They sent 68% (16/24) of the requested number of videos on average. The average score for PT care satisfaction was maintained at 87% in the experimental group (*P*=.99), whereas it decreased from 89% at 8 weeks to 74% at 24 weeks (*P*=.008) in the control group. App-related adverse events were not observed.

**Conclusions:**

The Hudl app/telehealth platform is feasible for delivering HEPs and maintaining HEP adherence in participants with chronic diseases who are experiencing financial distress.

**Trial Registration:**

ClinicalTrials.gov NCT02659280; https://clinicaltrials.gov/ct2/show/NCT02659280

## Introduction

### Background

Approximately 200 million Americans have at least one chronic disease, and 80 million have multiple chronic diseases [1-3]. Patients with chronic health conditions often must adhere to a long-term individualized home exercise program (HEP) to manage their symptoms and improve or maintain their cardiovascular health, flexibility, and/or strength [4]. Most HEPs are delivered on paper to be practiced at home, and accountability is checked at the next physical therapy (PT) visit [5]. However, low HEP adherence is common, especially when exercising is required over a longer period [4-6]. A recent meta-analysis in people with chronic health conditions supports this observation, as the authors reported only 33% full HEP adherence and 37% partial adherence, with no difference in adherence when follow-up was done face-to-face or over the phone [7]. Low HEP adherence is not entirely surprising as it is challenging for patients to stay on an HEP for an extended period. In fact, a study showed that patients start to slack in HEP adherence as early as after 2 weeks [8]. Low HEP adherence is a serious problem because it will compromise the improvement or stabilization of chronic health issues.

Exercise adherence is even more negatively impacted when patients have a lower socioeconomic status [9]. Patients experiencing financial distress do not have as many options to manage their chronic disease as do people with a higher socioeconomic status, who can visit a health club or hire a personal trainer. Consequently, the health situation of adults experiencing financial distress can worsen and eventually they may have to lean on resources from the health care system more often. This is a serious problem for the patients and for the health care system. Thus, it is critical for clinics and hospitals to find alternative options to reach and treat underserved patients with a challenging socioeconomic status, especially regarding the long-term management of chronic diseases. More specifically, there is a need to find an effective delivery method for HEP that (1) is more inspiring and satisfying to these patients so that they are motivated to keep practicing at home for an extended period [10] and (2) can be used as a tool to provide professional feedback on exercise correctness [5].

To achieve this goal in the long run, Medtronic Philanthropy funded our exploratory study, titled *Expanding Access to Physical Therapy for Underserved Patients with Chronic Disease*. In this study, we looked into apps on mobile phones or on touchscreen tablets to determine whether they could potentially be an effective tool to provide such feedback and motivation [5,11,12]. Regular feedback from a PT has shown to be essential to correctly execute the HEP [8,13]. However, to date, studies in which apps were used for rehabilitation provided only occasional phone support from a PT or used a virtual coach to motivate participants to perform the app-based exercises. These methods limit the extent to which individualized feedback can be provided [5,10,14]. To overcome this problem, we have chosen to use the free Hudl Technique app aiming to help patients with HEP adherence. This app, originally designed for athletes’ coaches, enables patients and the PT to record and share exercises on video; most importantly, it enables the PT to provide individualized feedback on the patients’ self-recorded videos by using overlaying audio and visual feedback cues [15]. An additional benefit of using the Hudl app as a part of telehealth (ie, providing and sending individualized feedback from a PT to a patient via the Hudl app) is that this part is reimbursable through Medicaid or similar health care coverage [16,17], thereby making it available to patients with lower socioeconomic status who are in need of follow-up and feedback on their long-term HEPs.

To date, the use of the Hudl app in health care has been limited because app-based rehabilitation approaches are relatively new. Only 3 studies have used the Hudl app in sports medicine such that the app was (1) used to demonstrate features for potential use in sports medicine [18]; (2) used as a training tool for athletes to avoid sport-related concussions [15]; or (3) found to be unreliable for 2D kinematic analysis to evaluate patellofemoral pain during running compared with a laboratory-based 3D analysis [19]. In fact, to the best of our knowledge, no studies have reported the use of the Hudl app for rehabilitation.

### Objectives

Therefore, we aimed to conduct an exploratory study in adults with chronic diseases who were experiencing financial distress to investigate whether the Hudl app combined with telehealth (experimental group) results in better HEP adherence than when HEP is delivered through the standard paper format (control group). We defined adults experiencing financial distress as those receiving Medicaid or similar health care assistance. Over 7 months, we sampled adults with chronic diseases who were experiencing financial distress by visiting a general outpatient PT clinic and recruited a total of 45 participants, 38 of whom were classified as having a neurological chronic disease (stroke, spinal cord injury, traumatic brain injury, Guillain-Barre syndrome, or Parkinson disease) and 7 with other types of chronic diseases, including heart disease, diabetes mellitus, low back pain, autoimmune disease, and chronic obstructive pulmonary disease.

Owing to the limited funding and recruitment setting, the recruitment results did not allow us to recruit sufficiently large sample sizes per specific diagnostic category to allow for separate subanalyses per diagnosis. Nonetheless, we decided to proceed with this study on the Hudl app with this patient pool for four reasons: first, as the Hudl app has not been used in research for rehabilitation purposes, it was essential to determine whether patients, especially those with neurological chronic diseases, were able to use the app. Second, it was important to determine whether patients with financial hardship could use the app when they may or may not have sufficient Wi-Fi access at home or may not always have a home. Third, it was important to determine whether patients would demonstrate long-term HEP adherence by using the app, beyond the initial period of enthusiasm for using a new tool. It should be noted that we followed up the participants for an extended period (ie, 24 weeks). Finally, if those aspects of feasibility were met, we needed to determine whether our patient pool collectively achieved better HEP adherence with the use of the app, compared with when exercises were provided in paper format. To address these four key components, we incorporated questions in our survey on the ease of use of the equipment, Wi-Fi access, HEP adherence, and overall telehealth and PT satisfaction as patient satisfaction has been linked to the success of treatment adherence [20-23]. We measured HEP adherence (ie, how often they practice HEP per week) and compliance in returning self-recorded videos weekly and sent out a survey at 8 and 24 weeks to inquire about their satisfaction with PT care and, for the experimental group, their satisfaction with the Hudl app/telehealth platform in terms of feasibility. The features of the Hudl app included videos of the physical therapist demonstrating the exercises and individualized feedback given by the physical therapist through digital features of the Hudl app projected on the self-recorded patient videos. We hypothesize that these unique features of the Hudl app would likely motivate the participants to stay in the program.

## Methods

### Recruitment

Between March 10, 2016, and November 5, 2017, we recruited participants through consecutive sampling who were discharged from the Hennepin County Medical Center and set to receive outpatient PT. Inclusion criteria were adults (≥18 years) who had a chronic disease and financial hardship defined as having Emergency Medical Assistance, Medical Assistance, Medicaid, Medicaid MCO (Medicare Contracted Organizations), hospital discount funding (*Hennepin Care*), or no insurance as those health care coverages are assigned only to adults experiencing financial distress. We assessed their ability to use a tablet device, internet features, and the Hudl app. Participants provided written informed consent. The study was approved by the institution’s internal review board (HSR#15-4040) and was performed in accordance with the Declaration of Helsinki.

### Study Design

Using a randomized controlled design, 60 sealed envelopes were used for 1:1 participant allocation. The treating therapist and data analyst were blinded to the allocation. Through electronic medical record review, we collected baseline data on age, sex, race, spoken language, health coverage status reflecting their financial situation, and diagnosis. The participant’s treating physical therapist designed and demonstrated the HEP during the first PT visit. All patients were instructed to perform exercises daily at home without any equipment.

**Figure 1:**
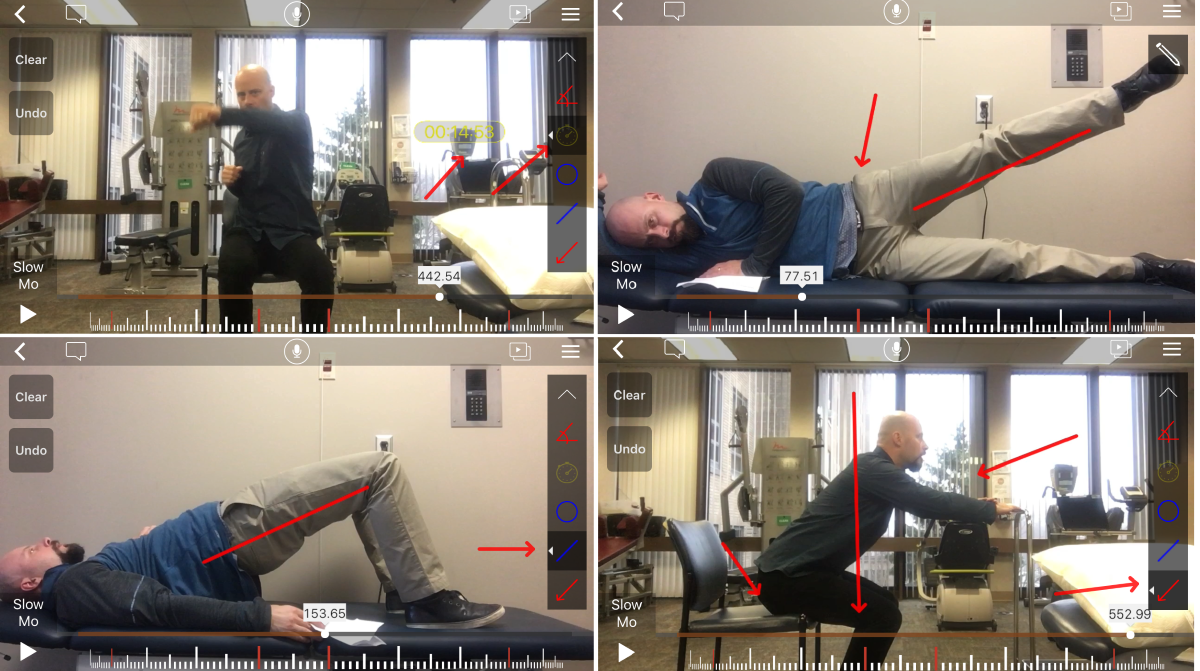
Examples of Hudl app video captures of the telehealth physical therapist’s home exercise program. The telehealth physical therapist used the tools displayed on the right-hand side of the screen (arrows, plumb line, chronometer, etc) to highlight the parts that participants needed to pay attention to.

The control group received individualized HEPs on paper and did not receive feedback. To maintain the same incentive for both groups, the control group also received a tablet but without the Hudl app installed. The participants in the experimental group received a tablet and were trained on using the Hudl app. The experimental group received weekly videos with individualized HEP (ranging between 4 minutes 37 seconds and 9 minutes 23 seconds), as demonstrated by the telehealth physical therapist. After reviewing the video of the telehealth physical therapist, the participants video recorded their HEP performance, showing at least one set of repetitions of all required exercises. In addition, they reported whether they had completed all the required sets. The required sets included 3 trials per set for all strengthening exercises (maximum 10 repetitions each) and 3 trials per set for all balance exercises (maximum 30 seconds each). The telehealth physical therapist reviewed the content and compliance to the HEPs and created a critiqued video review using the Hudl app tools (Figure 1). The most frequently used tools were (1) the *voice-over* to offer feedback; (2) the *chronometer* to record the time required to perform, for example, a single-limb stance test; (3) the *goniometer* to measure joint angles; (4) the *visual tool* displaying 2 videos side by side to show weekly progress or to compare the telehealth physical therapist’s video with the participant’s video, highlighting the differences in execution; (5) the *drawing tool* to review the participant’s postural symmetry during exercises; and (6) the *slow-motion video* to analyze dynamic balance and exercise sequences. The participants reviewed the critiqued video and, within a few days, received a new video from the telehealth physical therapist with a HEP for the following week. The participants only received a new video if they returned a video to a telehealth physical therapist.

At 8 and 24 weeks, participants completed a questionnaire anonymously on the web (17/45, 38%) or in paper format (28/45, 62%) if they had trouble completing the web-based survey. The survey asked the participants to quantify their average HEP adherence per week; overall PT satisfaction; and, for the experimental group, satisfaction with the app and telehealth use. Scoring for questions ranged from 1 (very dissatisfied or difficult) to 7 (very satisfied or easy). Overall PT satisfaction encompassed both the Hudl app with telehealth platform use and care or feedback from the telehealth physical therapist and treating PT service for the experimental group and HEP on paper and the treating PT service for the control group.

### Statistical Analysis

#### Power

The sample size was determined through a priori power analysis using G.Power 3.1 and based on data from a study with a similar setup, which reported 71% (SD 25%) training adherence in the group using a tablet versus 48% (SD 42%) in the group using a paper version [24]. With 85% power, *α*=.05, and a 1:1 enrollment ratio, we needed 16 participants per group. We aimed to recruit at least 5 additional persons per group to account for a 31% attrition rate, as reported in a previous study [24].

#### Data Analysis

Data were analyzed using JMP, version 13 (SAS Institute Inc, 1989-2007). The Shapiro-Wilk test informed the decision to conduct *t* tests versus Mann-Whitney *U* tests for between-group comparisons at 24 weeks or a paired *t* test versus Wilcoxon tests for within-group comparisons at 8 and 24 weeks. Nominal data were calculated using chi-square test for between-group comparisons [25] or with the McNemar test for within-group comparisons [26].

For the 7 questions on app with telemedicine use, we reported descriptively on the percentage of people who were unsatisfied (scores 1-3), neutral (score 4), or satisfied (scores 5-7). We used the McNemar test to evaluate differences in the ratio of people who were unsatisfied or neutral versus satisfied between 8 and 24 weeks. We reported on the proportion of people filling in the survey and the percentage of people satisfied with the PT treatment (scores 5-7). The average score (in percentage) given on overall PT satisfaction at 8 and 24 weeks was compared between and within groups.

We compared between- and within-group differences in percentage HEP adherence (based on the patient-reported number of sessions completed per week). We calculated the effect size for between-group differences in HEP adherence at 24 weeks (Hedges *g* for unequal sample sizes) [27] and repeated-measures effect size (*d_repmeas_*) [28] for within-group differences in HEP adherence [29].

We calculated the percentage of people returning videos and, of those participants, how many videos they returned compared with the requested weekly videos (reported in percentage).

## Results

### Demographics and Patient Characteristics

**Figure 2:**
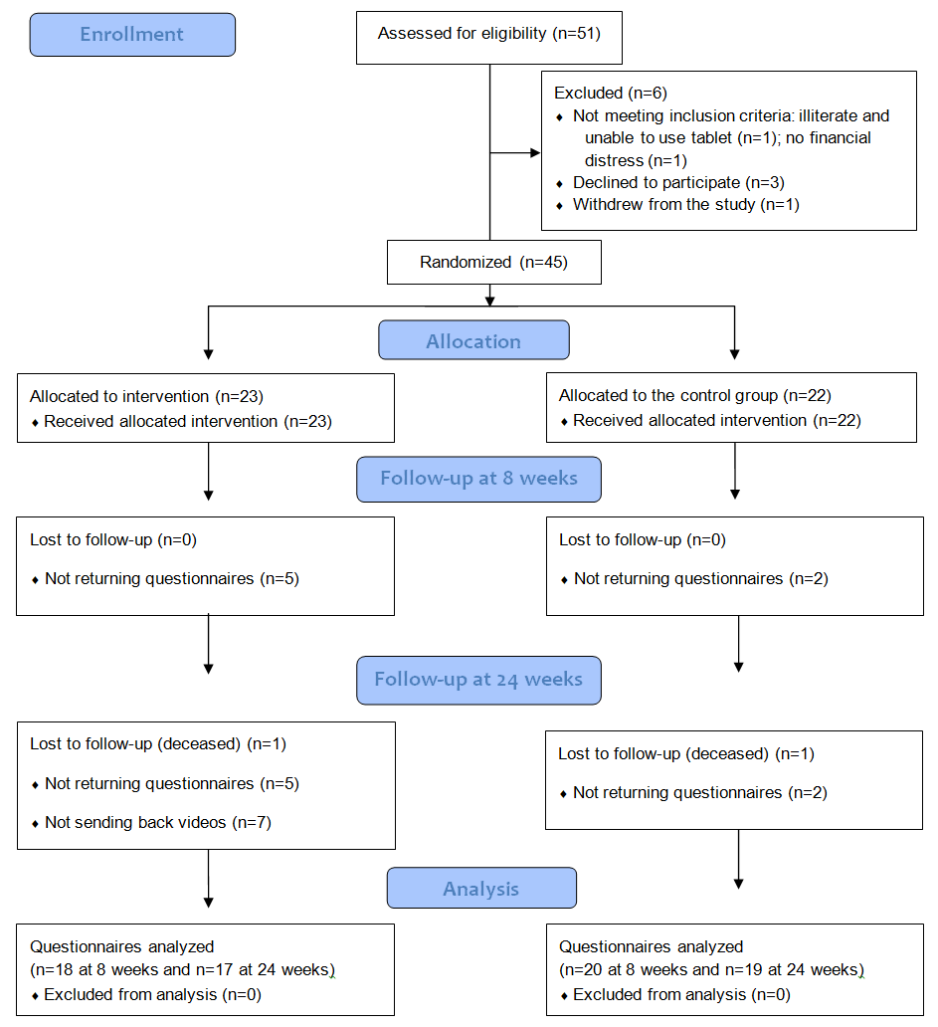
The CONSORT (Consolidated Standards of Reporting Trials) flowchart.

The CONSORT flowchart (Figure 2) illustrates the steps in the study. Of the 51 participants approached, 45 were randomized into experimental (n=23) and control (n=22) groups, and 17 and 19 participants completed the study, respectively. As stated earlier, of the 45 participants, 38 were classified as having a chronic neurological disease (stroke, spinal cord injury, traumatic brain injury, Guillain-Barre syndrome, or Parkinson disease) and 7 with other types of chronic diseases, including heart disease, diabetes mellitus, low back pain, autoimmune disease, and chronic obstructive pulmonary disease. Demographic (Table 1) and clinical (Table 2) data were reported for each group. There were no significant differences between the groups with respect to age (*U*=−0.72; *P*=.47), sex (*χ^2^*_1_=1.1; *P*=.30), race (*χ^2^*_1_=6.2; *P*=.29), or language (*χ^2^*_1_=5.3; *P*=.15). Table 2 details the type of chronic diseases the participants had and the duration of their chronic disease. Table 2 also mentions the type of individualized HEPs the participants received to manage their symptoms or impairments related to the chronic disease.

**Table 1:** Demographic data of the participants per allocated group (N=45).

Categories			Experimental group (n=23)		Control group (n=22)		Statistical tests^a^			*P* value	
Age (years), mean (SD)			47.26 (13.33)		44.59 (13.79)		*U*=−0.72			.47	
**Sex,** **n (%)**								*χ*^2^_1_=1.1			.30
	Male	16 (70)		12 (55)							
	Female	7 (30)		10 (45)							
**Race,** **n (%)**								*χ*^2^_1_=6.2			.29
	African	2 (9)		2 (9)							
	American Indian	0 (0)		1 (4)							
	Asian	1 (4)		0 (0)							
	Black	9 (39)		7 (32)							
	White	9 (39)		5 (23)							
	Latino	2 (9)		7 (32)							
**Language,** **n (%)**								*χ*^2^_1_=5.3			.15
	Amharic	1 (4)		0 (0)							
	English	21 (92)		16 (73)							
	Igbo	0 (0)		1 (4)							
	Spanish	1 (4)		5 (23)							

^a^Statistical tests: the chi-square test was used for sex, race, and language and the Mann-Whitney *U* test was used for age.

**Table 2:** Clinical data of the participants per allocated group (n=45).

Diagnosis	Experimental group (n=23)			Control group (n=22)			Type of home exercise program
	n (%)	Duration of symptoms^a^	n (%)		Duration of symptoms^a^		
Autoimmune disease	0 (0)	N/A^b^	1 (5)		3.5 months	Stretching, strengthening, and balance	
Chronic obstructive pulmonary disease	1 (4)	6 years and 2 months	0 (0)		N/A	Stretching and strengthening	
DM^c^	1 (4)	13 years and 8 months	1 (5)		20.5 years	Strengthening and balance	
DM and chronic low back pain	1 (4)	16 years and 9 months	1 (5)		5 years and 2 months for DM, 3 months for low back pain	Strengthening	
Guillain-Barre syndrome	0 (0)	N/A	1 (5)		3.5 months	Strengthening and assisted mobility	
Heart disease	1 (4)	7 years and 7 months	0 (0)		N/A	Stretching, strengthening, balance, and cardiorespiratory interval training	
Parkinson disease	1 (4)	4 months	0 (0)		N/A	Stretching, strengthening, and assisted mobility	
Spinal cord injury	3 (13)	Between 3.5 months and 20 years	2 (9)		Between 5 months and 17 years	Strengthening, cardiorespiratory training, assisted stretching, and balance	
Stroke	6 (27)	Between 3 and 5 months	9 (40)		Between 3 and 5.5 months	Strengthening, cardiorespiratory training, stretching, balance, assisted mobility, and functional activity	
Traumatic brain injury	9 (40)	Between 3 and 4 months	7 (31)		Between 3 and 5 months	Balance, strengthening, cardiorespiratory training, assisted stretching, and assisted mobility	

^a^For n=1, the duration is the exact duration of that person’s symptom. For n>1, the duration is the range of durations of the symptom.

^b^N/A: not applicable.

^c^DM: diabetes mellitus.

There were no app-related adverse events. Two participants died because of unrelated causes before the 24-week questionnaires were distributed. The electronic medical record stated that 1 participant (experimental group) died from natural causes, and the other participant (control group) had a fatal myocardial infarction.

### Primary Outcome

#### Satisfaction With PT Care and Use of the App and Telehealth in Terms of Its Feasibility

The average scores (in %) given on the overall PT experience in both groups were determined. There was no significant between-group difference at 24 weeks (*U*=342.5; *P*=.34). The within-group average score for PT satisfaction was maintained in the experimental group at 8 and 24 weeks (87%, SD 21%, to 87%, SD 19%; Wilcoxon *S*=−5.50; *P*=.99) but decreased significantly over time in the control group (89%, SD 24% to 74%, SD 31%; *S*=−62.00; *P*=.008).

**Figure 3:**
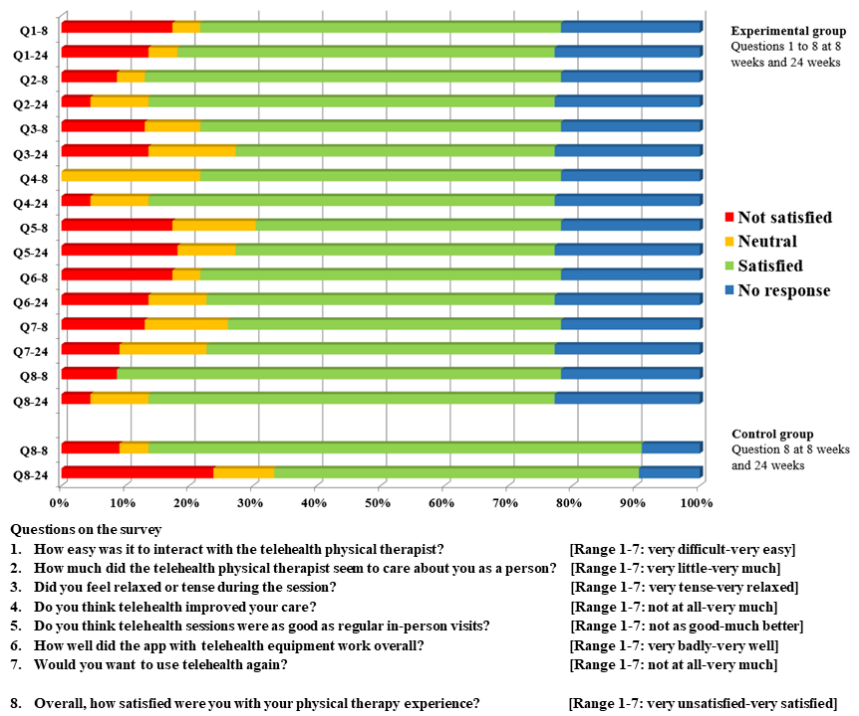
Percentage responses in categories unsatisfied (red), neutral (orange), satisfied (green), and no response (blue) from the survey completed by the experimental and control groups. The experimental group answered 7 questions related to app with telemedicine use, feasibility, and ease of interaction with the telehealth physical therapist. The eighth question was answered by both groups and pertained to their rating of satisfaction with the overall physical therapy experience. The answer options ranged from scores 1 to 3 (being not satisfied [scoring very unsatisfied to a little unsatisfied]), score 4 (neutral), or scores 5 to 7 (satisfied [scoring a little satisfied to very satisfied]) or no response. The question and answer options for each question are detailed at the bottom of the figure.

The percentage of people who were satisfied to very satisfied (scores 5-7) with the app and telemedicine use in terms of its feasibility is shown in Multimedia Appendix 1. There was no significant change in the ratio of people satisfied versus neutral or not satisfied between 8 and 24 weeks or between groups on the overall PT satisfaction question at 8 or 24 weeks. The percentage of people who were not satisfied (scoring 1-3, which meant *very unsatisfied* to *a little unsatisfied*), neutral (score 4), or satisfied (*a little satisfied* to *very satisfied*, scores 5-7) on all questions, including the overall PT satisfaction for both groups are shown in Figure 3.

#### HEP Adherence

Owing to the limited funding and recruitment setting, the recruitment results did not allow us to recruit sufficiently large sample sizes per specific diagnostic category to allow for separate subanalyses per diagnosis. Therefore, the experimental and control groups combined adults who all experienced financial distress but may have varied in terms of the diagnosis of chronic disease. The experimental group had an average HEP adherence of 4 (SD 2) times per week at 8 weeks to 5 (SD 2) times per week at 24 weeks (*d_repmeas_*=0.48). The control group decreased on average from 4 (SD 2) times per week to 3 (SD 2) times per week. The within-group differences in HEP adherence were not significant (experimental group *S*=27.5, *P*=.14; control group *S*=−46.5, *P*=.07), the latter reflecting a small effect size *d_repmeas_*=0.42. The between-group difference in HEP adherence at 24 weeks was statistically significant (*U*=2.49; *P*=.01), with a moderate effect size of Hedges *g*=0.76.

In total, 67% (12/18) and 82% (14/17) of participants in the experimental group at 8 and 24 weeks, respectively, were at least partially HEP adherent (ie, at least 4 times per week) compared with 70% (14/20) and 42% (8/19) at 8 and 24 weeks, respectively, in the control group. In the experimental group, 68% (15/22) of participants sent videos back, returning on average 68% of the requested number of videos (range 25%-100%). Participants did not report problems with Wi-Fi access at home; however, 43% (10/23) of the participants who used community-based Wi-Fi reported difficulty in returning videos because of occasional unreliable Wi-Fi. Participants who completed HEPs were 100% compliant with the requested number of trials per exercise and the requested number of exercises (verified through video recording).

## Discussion

### Overview

Given our patient pool of 45 adults experiencing financial distress, among which 38 had neurological chronic diseases and 7 had other types of chronic diseases described above, we explored whether the Hudl app with telehealth platform would be feasible in patients with physical and financial difficulties. If it proved to be feasible, we then asked if participants who were using the Hudl app with telehealth platform were able to maintain their HEP adherence over 24 weeks and whether the HEP adherence in that group would be higher than that in a control group that used HEP on paper. We specifically selected participants who were experiencing financial distress because our long-term goal is to provide a cost-effective solution to patients in a lower socioeconomic status to manage their health effectively, which, in turn, will lead to significant cost savings for the health care system. This is the first critical step for achieving such a goal.

### Principal Findings

We obtained the following answers to whether the Hudl app with telehealth platform is feasible: (1) participants were able to use the app. (2) None of the participants reported problems with Wi-Fi at home; however, 43% (10/23) of the participants who used community-based Wi-Fi reported some difficulty returning videos because of occasional unreliable Wi-Fi. (3) Participants who used the app with telehealth platform maintained their HEP adherence over 24 weeks. This period of HEP adherence was long enough so that any initial excitement of the novelty should have worn off over time, revealing the real feasibility of the app/telehealth combination approach over this prolonged period. (4) There was a statistically significant difference in HEP adherence between both groups at 24 weeks because participants who used the app with telehealth maintained their HEP adherence whereas participants in the control group decreased in HEP adherence. These results suggested the overall feasibility of the app/telehealth combination approach.

In addition, as mentioned in the Introduction section, patient satisfaction is an important aspect of feasibility, linked to the success of treatment adherence, and is an important outcome for quality of health care [20-23]. Care and ease of interaction with the physical therapist have been shown to be rated of the highest importance for patient satisfaction [30]. The satisfaction with PT care dropped significantly in the control group from 8 to 24 weeks. In contrast, we observed that patient satisfaction was rated high for the group using the app and telerehabilitation, with one of the highest satisfactions being the *care of the telehealth-PT* through 8 to 24 weeks. We believe that the high satisfaction score was attributed to the specific feedback given through the various app tools, which were superimposed on the patient’s self-recorded video. 

In summary, this study indicated the feasibility of the combined app with telehealth approach in a specific group of adults who experienced financial hardship. The study showed promising results in terms of HEP adherence for symptoms of participants who had mostly neurological chronic diseases. These promising results justify the validation of this study in a larger cohort to demonstrate the cost-effectiveness and efficacy of the app with telehealth in terms of health outcomes in populations with specific chronic diseases; therefore, these app with telehealth platforms can be implemented more widely. Implementation of such strategies in health care would benefit patients because of reduced costs in involved expenses, including transportation. In addition, during pandemic periods with requirements for social distancing or quarantine for high-risk adults, our combined app/telehealth approach might offer a valuable alternative to monitor the status of patients who need long-term follow-up care [31].

### Limitations

As mentioned earlier, our patient pool does not have a sufficient sample size and spectrum of chronic diseases to allow us to perform subanalyses per diagnosis group. Therefore, we cannot make any assumptions whether adults with one specific type of chronic disease would have better HEP adherence with the use of the Hudl app with telerehabilitation than another type. This issue should be addressed in future studies.

### Comparison With Previous Work

A total of 3 other studies have reported HEP adherence with an app in adults with a frozen shoulder, diseases involving the musculoskeletal system, and Parkinson disease [5,10,14]. The studies in adults with a frozen shoulder or Parkinson disease used custom software for their apps [5,14], whereas the study with the musculoskeletal system used an app connected to free exercises on the web [10]. Participants either did not receive feedback at all [5], received only periodic phone calls or motivational text messages [10], or interacted only with a virtual coach for 5 minutes per exercise session [14]. In all the 3 studies, the results were reported over 3 to 4 weeks only, which poses a potential concern in terms of ascertaining whether participants would keep practicing for the required long-term duration that goes with managing chronic symptoms. Thus, it is possible that the excitement of the novelty of the app might have motivated the participants to follow the program during that time, resulting in relatively high HEP adherence. In contrast, we monitored HEP adherence for an extended period (ie, 24 weeks).

The HEP adherence in our experimental group was 67% (12/18) and 82% (14/17) partial HEP adherence at 8 and 24 weeks, respectively. Although a direct comparison is not possible because of the difference in sample size, the adherence percentages were higher than those reported in a recent meta-analysis in which 37% partial HEP adherence was reported in 11 studies, with a total of 1231 adults with chronic diseases [7]. These studies used traditional approaches of delivering HEP on paper, and their results were similar even when provided in different settings—in a center, off-site, or at home—and irrespective of whether they received phone follow-up at home [7].

### Conclusions

The results from this study indicate that the use of the Hudl app with telehealth platform is feasible for participants experiencing financial distress and demonstrate that participants are able to maintain a high level of HEP adherence when using the app compared with when HEP is delivered on paper. Our long-term goal is to provide a cost-effective solution to adults with lower socioeconomic status to manage their health more effectively in the long run in such a way that they can benefit from reimbursement through Medicaid or a similar health care coverage. This project is the first critical step for such a goal, with promising results justifying the planning of a larger sample clinical trial.

## Appendix

Multimedia Appendix 1 Percentages of satisfaction scores (scores 5-7) using the Hudl Technique coaching app in terms of its feasibility.

Multimedia Appendix 2 CONSORT-EHEALTH checklist (V 1.6.1).

## Abbreviations

HEP: home exercise program
PT: physical therapy
