# Supplementary material for: Combining a Hudl App With Telehealth to Increase Home Exercise Program Adherence in People With Chronic Diseases Experiencing Financial Distress: Randomized Controlled Trial
Source: JMIR Form Res. 2021 Mar 18;5(3):e22659. doi: 10.2196/22659 (PMC8075043; doi:10.2196/22659)
Supplement: Multimedia Appendix 1 [file formative_v5i3e22659_app1.docx]

**Multimedia Appendix 1.** Percentage of satisfaction scores (scores 5-7) using the Hudl Technique coaching app in terms of its feasibility.

| Questionnaire: percentage of people being satisfied (scores 5-6) to very satisfied (maximum score 7) on the questions asked^a^ | Experimental group^e^, n (%) | | Statistical tests^b^ | |
| --- | --- | --- | --- | --- |
|  | 8 weeks (n=18) | 24 weeks (n=17) | chi-square or McNemar test (df=1) | *P* value |
|  |  |  |  | |
| 1. Easiness of interaction with telehealth physical therapist | 13 (72) | 13 (76) | .3 | .62 |
| 2. Care of the telehealth physical therapist | 15 (83) | 14 (82) | .0 | *>*.99 |
| 3. Relaxation during session | 13 (72) | 11 (65) | .3 | .62 |
| 4. Did telehealth improve your care? | 13 (72) | 14 (82) | .0 | *>*.99 |
| 5. Telehealth comparable with in-person visits | 11 (61) | 11 (65) | .5 | .48 |
| 6. Telehealth equipment: How well did it work? | 13 (72) | 12 (71) | .0 | *>*.99 |
| 7. Would you use telehealth again? | 12 (67) | 12 (71) | .3 | .62 |
| Total satisfaction score with PT^c^ care (HEP^d^, app with telehealth) | 16 (89) | 14 (82) | .8 | .37 |
|  | Control group^e^, n (%) | | Statistical tests^b^ | |
|  | n=20 | n=19 | chi-square or McNemar test (df=1) | *P* value |
| Total satisfaction score with PT care (HEP), n (%) | 17 (85) | 12 (63) | 2.3 | .13 |
|  |  |  |  | |

^a^Scoring attribution: 1 (worst outcome, eg, very dissatisfied, very difficult) to 4 (neutral) to scores 5 to 7, with 5 (eg, satisfied, easy) and score 7 as the best outcome (eg, very satisfied, very easy).

^b^Statistical tests: the chi-square test for between-group differences; the McNemar test for within-group differences.

^c^PT: physical therapy.

^d^HEP: home exercise program.

^e^Between-group comparison at 8 weeks: *χ^2^_(1)_*=.1; *P*=.72; and at 24 weeks: *χ^2^_(1)_*=1.7; *P*=.20
